# Behavioural reconsolidation interference not observed in a within-subjects design

**DOI:** 10.1038/s41539-022-00143-w

**Published:** 2022-10-11

**Authors:** Michael Batashvili, Rona Sheaffer, Maya Katz, Yoav Doron, Noam Kempler, Daniel A. Levy

**Affiliations:** Baruch Ivcher School of Psychology, Reichman University, Herzliya, Israel

**Keywords:** Consolidation, Human behaviour, Forgetting

## Abstract

Studies of reconsolidation interference posit that reactivation of a previously consolidated memory via a reminder brings it into an active, labile state, leaving it open for potential manipulation. If interfered with, this may disrupt the original memory trace. While evidence for pharmacological reconsolidation interference is widespread, it remains unclear whether behavioural interference using the presentation of competing information can engender it, especially in declarative memory. Almost all previous studies in this area have employed between-subjects designs, in which there are potential confounds, such as different retrieval strategies for the multiple conditions. In the current studies, within-subjects paradigms were applied to test the effects of reconsolidation interference on associative recognition and free recall. In Experiment 1, participants engaged in pair-associate learning of unrelated object pictures on Day 1, and after a reminder, interference, reminder + interference, or no manipulation (control) on Day 2, were tested on associative recognition of these pairs on Day 3. In Experiments 2 and 3, memoranda were short stories studied on Day 1. On Day 2, stories were assigned to either control, reminder, interference by alternative stories, or reminder + interference conditions. On Day 3 participants recalled the Day 1 stories, and answered yes/no recognition questions. Reminders improved subsequent memory, while interference was effective in reducing retrieval in differing degrees across the experiments. Importantly, the reminder + interference condition was no more effective in impairing retrieval than the interference-alone condition, contrary to the prediction of the behavioural reconsolidation-interference approach.

## Introduction

Processes of memory consolidation may lead to strengthening of newly formed representations through plasticity processes, but consolidated memories may be vulnerable to manipulations enabling changes in the memory over time^1^. Memory reconsolidation theory posits that even previously consolidated and stable memories can be made labile by reactivating the memory. When the memory is in this unstable stage it can be modified, or even erased^2^. Only if the memory reconsolidates does it return to relative stability.

To date, the most striking demonstrations of memory-modifying interventions leading to post-activation erasure rather than to reconsolidation have been the ability of pharmacological interventions to abolish fear conditioning in animals^3^. However, such processes have also been found in humans. Administration of a beta blocker (e.g., propranolol) has been shown to block protein synthesis that had demonstrated to be specifically critical for emotional memory reconsolidation^4^, for example, in PTSD^5–7^. These studies typically find that the reconsolidation process of the original fear memory is impaired, thereby interrupting fear response mechanisms. However, there is also evidence that pharmacological reconsolidation interference is not always obtained both in animals^8^ and humans^9,10^, and it has been shown that there is a publication bias in the literature towards presenting evidence of reconsolidation interference^11^.

Nevertheless, there is a growing body of research claiming that reconsolidation interference can be induced by a behavioural manipulation without the need for pharmacological intervention (see^12^ for a review). For example, James and colleagues^13^ showed that playing a visuospatial computer game (Tetris) reduced the amount of traumatic intrusive memories after participants had watched videos of traumatic events. In their paradigm, 24 h after watching the traumatic videos, the memories were reactivated by showing stills from the video clips and then, following a 10-minute gap, participants played the computer game. Over the following seven days, those participants had fewer intrusions from the trauma film than participants who had not played Tetris, suggesting that the visuospatial interference task after the reactivation of the trauma memory (making it labile), interfered with the reconsolidation of this memory. This theory has been supported by other studies, showing the possibility that behavioural interference methods may lead to reconsolidation interference in human participants^14,15^. However, there is also evidence showing that behavioural reconsolidation interference is not readily observed in both between-subjects^16^ and within-subjects^17^ designs.

One of the main issues with previous research in this area is that the majority of studies that show evidence of reconsolidation interference have used between-subjects’ paradigms, which allows for potential confounds, such as individual differences in retrieval strategies across groups, potentially accounting for the significant effects found. For example, in these studies groups differ in whether they receive a reminder of the target memory leading to activation, an interfering stimulus, both, or neither. Participants who receive a reminder only may utilize a retrieval strategy based on consistent utilization of the reminder to retrieve target information, whereas those in other conditions cannot or are less likely to do so, such that group differences might not be indicative of memory erasure or modification. The between-subjects design of the study by Sinclair and Barense^18^ avoids this concern by manipulating the order of the reactivation and interference phases for all participants. However, this is not the case for other studies where participants are split into separate groups receiving either a reminder, interference, none, or both.

Some researchers have attempted to apply a within-subjects manipulation within mixed design studies^19–21^. However, the reconsolidation effects reported are limited to the between-subjects aspects of the studies^22^. Therefore, to examine whether these effects could be seen using a purely within-subjects design, Levy and colleagues conducted a series of experiments focusing on episodic reconsolidation interference in recognition memory for object pictures^22^. Those experiments were conducted over three days, to establish memories consolidated during overnight sleep, and an additional night’s sleep to enable reconsolidation after interventions. The studies used four separate conditions: reminder, interference, combined condition (reminder and interference), and control (encoding only). On day one, participants encoded a set of object pictures. On day two, the manipulations were employed; for example, in the reminder condition, participants are presented with a reminder (e.g., given the names of a portion of the pictures and asked to rate how well they remember the actual picture). For the interference task, participants are presented with pictures not encoded on day one, and asked to rate the object pictures for liking, symmetry and colour. The reminder and interference condition combined both aspects of the previous two conditions, whereby a reminder is first used, shortly followed by an interference. For the control condition, participants are not shown anything during the second day. Finally, on day three participants performed a two-alternative forced choice recognition task where they were presented with two pictures, the picture they saw on day one and another picture of the same object (but a different size, colour or shape) they had not seen at all, and their recognition accuracy and RT were measured. According to the claim that reconsolidation interference may be engendered by making memory representations labile and more susceptible to interference through the provision of a reminder prior to the interfering stimulus, poorer memory is expected in the reminder + interference condition than in the interference-only condition.

These experiments yielded no such evidence of reconsolidation interference. This was the case over all three studies, even when changes were made to increase the chances of eliciting the effects, e.g., ensuring that within the combined reminder and interference condition the presentation of the reminder and interference had minimal time between presentations, or weakening the reminder. These results seem to indicate that when care is taken to control of the effects of manipulations on retrieval strategies, and memory changes are assessed via success in recognition of experienced stimuli rather than by intrusions of stimuli studied in other stages of the experiment, behavioural reconsolidation interference is not easily demonstrated.

Levy and colleagues^22^ noted several possible explanations for the absence of the expected reconsolidation interference effects. That could have been due to the inordinate strength of the reminder in comparison to the interference; however, reminder strength has been shown to be unimportant in the generation of reconsolidation interference effects^23^. Levy and colleagues^22^ also noted that the opposite is possible; using the names of the objects, within the reminder condition, might not have been substantial enough to reactivate the encoded memories in order to make them labile.

To achieve greater clarity regarding the question of whether reconsolidation interference can indeed weaken consolidated memories, further investigation of the possible boundary conditions of that process are warranted. Aside from the issue of reminder strength and interference strength, it is possible that behavioural reconsolidation interference might not be sufficiently salient in recognition memory for individual pictures, in which simple item familiarity may be used to discriminate studied and unstudied stimuli. Therefore, in the current studies we turned to tests of associative memory and complex free recall. In all these studies, evidence for reconsolidation interference would be provided by poorer memory for studied materials when a reminder is provided before interference than for interference without a prior reminder^13–15,22^.

## Experiment 1

### Results

Experiment 1 employed a test of associative recognition memory for pairs of pictures. Using this paradigm, the strength of the encoding can be increased by instructing participants to create deep-encoding associations that will serve as effective reminders^24,25^ for reactivating the target memory into a labile state. Additionally, we used the classic A–B, A–C retroactive interference paradigm, the standard paradigm for engendering memory interference^26^. Furthermore, we asked participants to perform Remember/Know judgments following their recognition judgments, to provide an assay of the effects of the putative reconsolidation interference manipulation on recollection strength^22^. We hypothesized that under these conditions, it might be possible to observe reconsolidation interference even within-subjects. To assess the effects of reminder and interference manipulations on accuracy (percentage correct), reaction time (ms), and self-reported ‘remember’ responses (percentage), three 2 (reminder/no reminder) × 2 (interference/no interference) repeated measures ANOVAs were conducted.

#### Accuracy

**Fig. 1:**
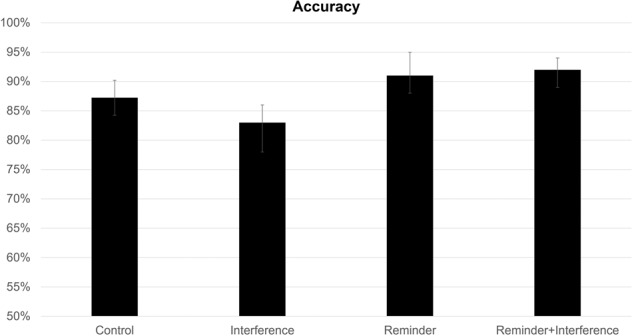
Accuracy (%) for each condition of Experiment 1. Brackets represent 95% confidence intervals.

For accuracy, there was a significant main effect of reminder, *F*(1,39) = 29.43, *p* < 0.001, η² = 0.22, BF_10_ = 0.75, showing that participants had greater accuracy for pairs where a reminder was employed (Mean = 92%, SD = 0.09%) compared to pairs that had no reminder (Mean = 85%, SD = 0.11%). There was also a significant main effect of interference, *F*(1,39) = 6.81, *p* = 0.01, η² = 0.02, BF_10_ < 0.001, showing that participants had poorer accuracy for pairs when an interference was employed (Mean = 0.87%, SD = 0.14%) compared to pairs that had no interference (Mean = 0.89, SD = 0.11; see Fig. 1). There was a marginally significant interaction found between reminder and interference, *F*(1,39) = 3.39, *p* = 0.07, η² = 0.03, BF_10_ = 0.68. Post-hoc paired samples t-tests were calculated to explore differences between the four conditions (see Table 1). These indicated that memory for stimuli in the reminder + interference condition was significantly superior to memory in the interference-alone condition, the opposite of what would be expected if reminders engender reconsolidation interference.

**Table 1:** Post-hoc paired samples t-tests comparing conditions on accuracy (with Cohen’s d effect size and Bayes factors included).

Comparison	*t* value (df)	*p* value	Cohen’s *d*	BF_10_
Control – Interference	2.90 (39)	0.006^a^	0.37	6.22
Control – Reminder	−2.27(39)	0.029	0.36	1.68
Control – Reminder + Interference	−3.05 (39)	0.004^a^	0.37	8.72
Interference – Reminder	−5.92 (39)	<0.001^a^	0.87	25045.43
Interference – Reminder + Interference	−5.62 (39)	<0.001^a^	0.84	10142.82
Reminder – Reminder + Interference	−0.11 (39)	0.916	0.02	0.17

^a^Significant comparison after Bonferroni correction.

#### Response times

**Fig. 2:**
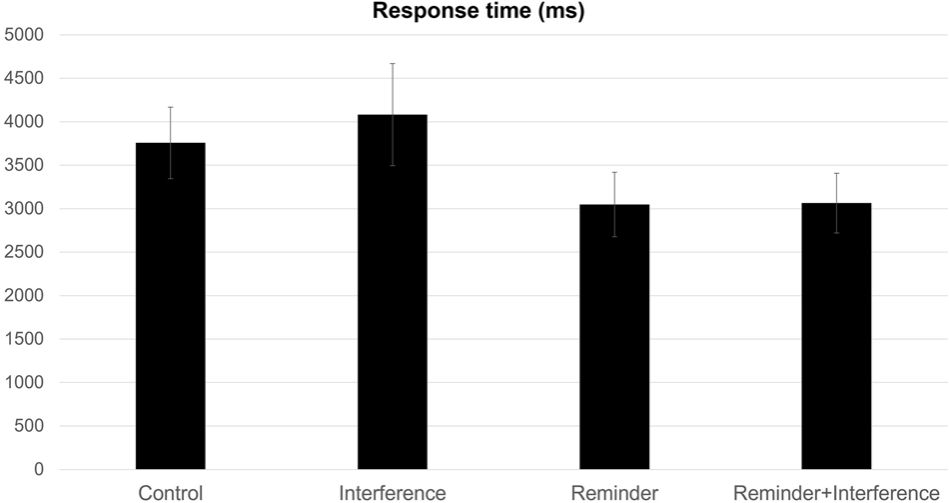
Reaction time (ms) for each condition of Experiment 1. Brackets represent 95% confidence intervals.

For RT, there was a significant main effect of reminder, *F*(1,39) = 28.95, *p* < 0.001, η² = 0.32, BF_10_ = 1.00, showing that participants had faster reaction time for pairs where a reminder was employed (Mean = 3055 ms, SD = 1116 ms) compared to pairs that had no reminder (Mean = 3919 ms, SD = 1562 ms). There was also a significant main effect of interference, *F*(1,39) = 4.455, *p* = 0.04, η² = 0.01, BF_10_ < 0.001, showing that participants had a slower reaction time for pairs when an interference was employed (Mean = 3572 ms, SD = 3572 ms) compared to pairs that had no interference (Mean = 3402 ms, SD = 3402 ms; see Fig. 2). There was a nearly significant interaction found between reminder and interference conditions, *F*(1,39) = 3.63, *p* = 0.06, η² = 0.01, BF_10_ = 0.45. Post-hoc paired samples t-tests were calculated to explore differences between the four conditions (see Table 2). In this measure as well, the comparisons indicated that correct associative recognition of stimuli in the reminder + interference condition was significantly faster than in the interference-alone condition, the opposite of what would be expected if reminders engender reconsolidation interference.

**Table 2:** Simple effects analyses comparing conditions on reaction time (with Cohen’s d effect size and Bayes factors included).

Comparison	*t* value (df)	*p* value	Cohen’s *d*	BF_10_
Control – Interference	−2.31 (39)	0.026	0.25	1.80
Control – Reminder	5.21 (39)	<0.001^a^	0.55	3004.97
Control – Reminder + Interference	4.42 (39)	<0.001^a^	0.54	312.57
Interference – Reminder	5.18 (39)	<0.001^a^	0.86	2725.18
Interference – Reminder + Interference	4.75 (39)	<0.001^a^	0.55	786.22
Reminder – Reminder + Interference	−0.21 (39)	0.837	0.01	0.17

^a^Significant comparison after Bonferroni correction.

#### Remember/Know judgments

**Fig. 3:**
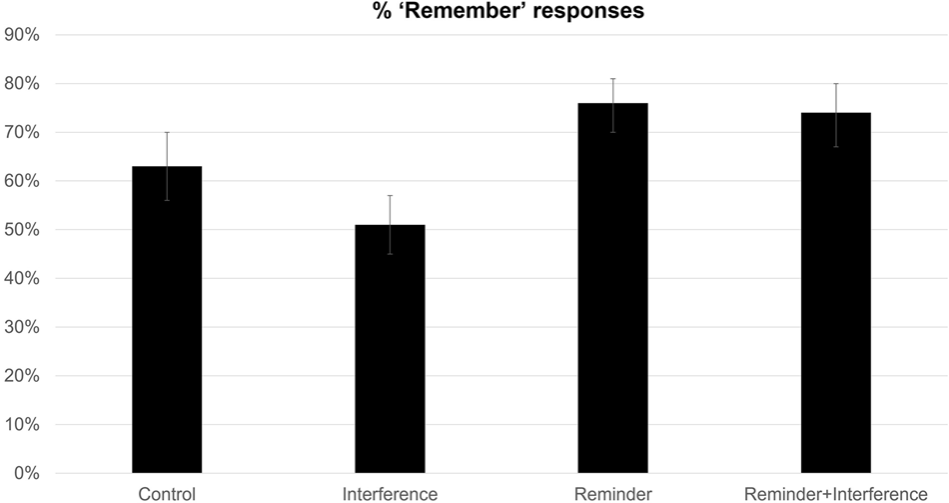
Percent ‘Remember’ responses for each condition of Experiment 1. Brackets represent 95% confidence intervals.

For Remember/Know judgments, there was a significant main effect of reminder, *F*(1,39) = 30.09, *p* < 0.001, η² = 0.22, BF_10_ = 0.01, showing that participants gave more “Remember” responses when there was a reminder present (Mean = 74.54%, SD = 18.46%) compared to pairs that had no reminder (Mean = 57.26%, SD = 20.83%). There was also a significant main effect of interference, *F*(1,39) = 35.71, *p* < 0.001, η² = 0.02, BF_10_ < 0.001, showing that participants gave fewer “Remember” responses when interference was employed (Mean = 62.35%, SD = 19.55%) compared to pairs that had no interference (Mean = 69.50%, SD = 19.74%; see Fig. 3). Finally, there was a significant interaction found between reminder and interference conditions, *F*(1,39) = 9.753, *p* = 0.003, η² = 0.03, BF_10_ = 0.36. Post-hoc paired samples t-tests were calculated to explore this interaction further and explore differences between the four conditions (see Table 3). As for the previous assays, recollection strength (as expressed in percentage of correct recognitions assigned a “Remember” response) in the reminder + interference condition was significantly greater than in the interference-alone condition, unlike what would be expected if reminders engender reconsolidation interference.

**Table 3:** Simple effects analyses comparing conditions on percent ‘Remember’ reports (with Cohen’s d effect size and Bayes factors included).

Comparison	*t* value (df)	*p* value	Cohen’s *d*	BF_10_
Control – Interference	5.38 (39)	<0.001^a^	0.54	50004.09
Control – Reminder	−3.80 (39)	<0.001^a^	0.55	56.82
Control – Reminder + Interference	−2.99 (39)	0.005^a^	0.46	7.65
Interference – Reminder	−8.17 (39)	<0.001^a^	1.41	>10^6^
Interference – Reminder + Interference	−6.62 (39)	<0.001^a^	1.14	201095.37
Reminder – Reminder + Interference	1.15 (39)	0.256	0.12	0.32

^a^Significant comparison after Bonferroni correction.

### Discussion

For object-picture associative recognition memory judgments, reminders were effective in improving accuracy, shortening RTs, and increasing the proportion of ‘Remember’ responses relative to a baseline condition. In contrast, retroactive interference by additional association formation impaired accuracy, lengthened RTs, and decreased the proportion of ‘Remember’ responses relative to a baseline condition. The key findings of this experiment are that despite the fact that both those manipulations were effective, providing reminders before interference did not weaken memory relative to interference-only in any of the three assays.

These accuracy and RT results converge with our prior reports of behavioural reminder + interference manipulations not yielding reconsolidation interference on item recognition memory^22^. However, it is still possible that the lack of reconsolidation interference effects in this case as well could be due to the recognition aspect of the study being insufficiently challenging to participants. There is some evidence that for picture pairs, associative familiarity might be mobilized to make recognition judgments, even in the absence of meaning-based unitization^27^. However, given that the manipulations employed yielded both reminder strengthening and interference weakening, and that performance was well below ceiling, the interaction of those effects posited by the behavioural reconsolidation interference account should have been manifest in accuracy measures to some degree, since recollection also contributes to recognition. Similarly, that explanation would not account for the lack of impact of the reminder + interference manipulation on ‘Remember’ judgments, which are asserted to reflect recollection^28^.

There are two additional considerations that might be responsible for the absence of reconsolidation interference effects observed in this experiment. One is that the disruption of prior representations engendered by reminders followed by interference is simply not powerful enough to erase even recollective aspects of recognition memory. It is conceivable that only when memory is assessed by free recall would the specific mnemonic disruption posited to follow reminder-potentiated interference emerge. Secondly, associative memory for object pictures in the format utilized in this study is not extremely ecological^15^. It is possible that the effects of reconsolidation interference on declarative memory would emerge for more complex and structured mnemonic constructs, in which across-stimulus associations might be more susceptible to lability induced by reminders and therefore more vulnerable to interference. That episodic complexity characterizes everyday experience, and might also be found in a narrative story. Reconsolidation-interference effects have been shown in previous research using narrative stories as stimuli, but have only been used in between-subjects designs^14,15^. We therefore conducted an additional experiment using narrative materials.

## Experiment 2

### Results

To address the possible lack of susceptibility of recognition memory judgments to modulation of interference by reminders, whilst also trying to relate the stimuli to experiences that participants may come across in their ‘real’ life, plausible narrative stories were created and used as stimuli. Memory for such stories following the four relevant intervention conditions was assessed. To examine the effects of reminder and interference manipulations on recall and recognition scores, two 2 (reminder/no reminder) x 2 (interference/no interference) repeated measures ANOVAs were conducted.

#### Recall

For recall, there was a significant main effect of reminder, *F*(1,51) = 15.89, *p* < 0.001, η² = 0.08, BF_10_ = 1.00, showing that participants had greater recall for pairs where a reminder was employed (Mean = 5.40, SD = 4.75) compared to pairs that had no reminder (Mean = 3.75, SD = 4.48). There was no significant main effect of interference, *F*(1,51) = 0.20, *p* = 0.66, η² = 0.001, BF_10_ = 0.003. There was also no significant interaction found for reminder and interference conditions on recall accuracy, *F*(1,51) = 0.81, *p* = 0.37, η² = 0.006, BF_10_ = 0.17. (see Table 4 for recognition means and SDs). An exploratory post-hoc paired samples *t* test, with Bonferroni correction applied, was used to directly compare the reminder and reminder + interference condition but was shown to be non-significant, *t*(51) = 0.895, *p* = 0.375, *d* = 0.14.

**Table 4:** Recall accuracy mean and SD scores in Experiment 2.

Condition	Mean	SD
Control	3.88	4.68
Interference	3.62	4.28
Reminder	5.08	4.74
Reminder + Interference	5.73	4.76

#### Recognition

For recognition, there was no significant main effect of reminder, *F*(1,51) = 0.67, *p* = 0.42, η² = 0.004, BF_10_ = 0.20, nor of interference, *F*(1,51) = 1.66, *p* = 0.20, η² = 0.01, BF_10_ = 0.33, and no significant interaction found for reminder and interference conditions on recognition accuracy, *F*(1,51) = 0.03, *p* = 0.86, η² < 0.001, BF_10_ = 0.07 (see Table 5 for recognition means and SDs).

**Table 5:** Recognition accuracy mean and SD scores in Experiment 2.

Condition	Mean	SD
Control	10.79	2.15
Interference	10.38	2.41
Reminder	10.96	2.31
Reminder + Interference	10.65	2.30

### Discussion

In this free recall paradigm, reduced recall accuracy for the reminder + interference combined condition was not observed. In fact, recall accuracy was numerically highest for this condition. However, this must be considered with caution, as we did not find a main effect of interference, and so the interactive effect might not have emerged due to the general lack of effectiveness of interference. As can be seen in Table 4, recall accuracy for the interference condition was similar to the control condition, indicating that the interference may not have been strong enough, perhaps because participants might not have thought of the interference story as being similar enough to the original story. Additionally, whilst research has noted that the strength of the reminder may be unimportant to generate reconsolidation interference^23^, it was thought that the reminder may have caused participants to think about the story more than necessary. Finally, some participants’ recall was zero for some of the stories (not specific to any one story).

Considering these three issues, in an additional study the paradigm was changed slightly regarding interference and reminder techniques in an attempt to increase the chances of identifying reconsolidation interference.

## Experiment 3

### Results

As in Experiment 2, memory for originally studied stories was assessed after interventions yielding the four interactive conditions of reminders and interference, with modifications made vis à vis the prior experiment intended to make the reminder more focused and provide stronger interference. To examine the effects of reminder and interference manipulations on recall and recognition scores, two 2 (reminder/no reminder) x 2 (interference/no interference) repeated measures ANOVAs were conducted.

#### Recall

**Fig. 4:**
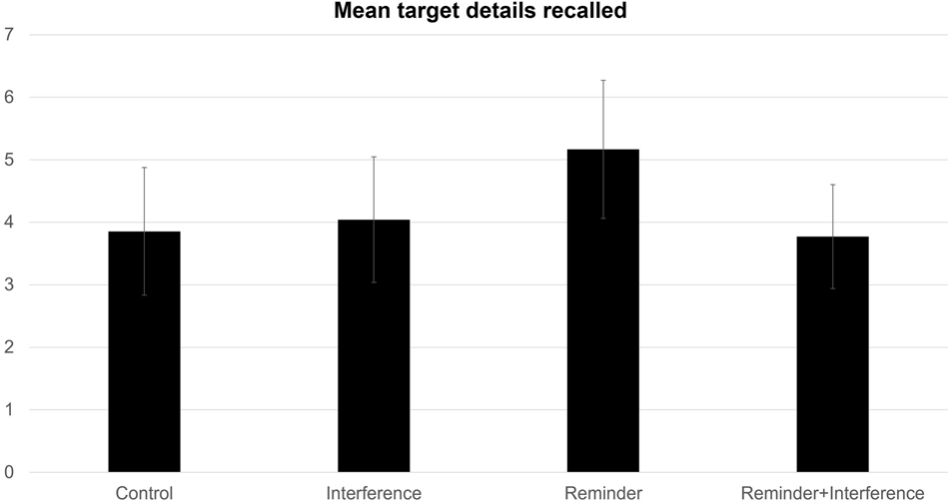
Recall accuracy for each condition of Experiment 3, in mean number of correctly recalled details of 25 possible target details. Brackets represent 95% confidence intervals.

There was no significant main effect of reminder, *F*(1,47) = 1.45, *p* = 0.23, η² = 0.01, BF_10_ = 0.33, or interference factors, *F*(1,47) = 2.12, *p* = 0.15, η² = 0.02, BF_10_ = 0.43, on recall accuracy. However, there was a significant interaction between reminder and interference effects on recall accuracy, *F*(1,47) = 5.06, *p* = 0.03, η² = 0.03, BF_10_ = 0.15 (see Fig. 4). Simple effects analyses were conducted showing that significant differences were only found between the reminder condition and all other conditions (see Table 6); however, after applying the Bonferroni correction, none of the comparisons fell below the adjusted alpha to be considered significant.

**Table 6:** Post-hoc paired samples t-tests comparing conditions on accuracy scores in Experiment 3 (with Cohen’s d effect size and Bayes factors included).

Comparison	*t* value (df)	*p* value	Cohen’s d	BF_10_
Control – Interference	−0.40 (47)	0.69	0.05	0.17
Control – Reminder	−2.30 (47)	0.03	0.38	1.70
Control – Reminder + Interference	0.14 (47)	0.89	0.02	0.16
Interference – Reminder	−1.83 (47)	0.07	0.30	0.73
Interference – Reminder + Interference	0.50 (47)	0.62	0.08	0.18
Reminder – Reminder + Interference	2.29 (47)	0.03	0.37	1.68

#### Recognition

As far as recognition accuracy (yes/no questions), there was no significant main effect of reminder, *F*(1,47) = 0.02, *p* = 0.88, η² = <0.001, BF_10_ = 0.15, or of interference factors on recognition accuracy, *F*(1,47) = 1.53, *p* = 0.22, η² = 0.01, BF_10_ = 0.38. There was also no significant interaction between reminder and interference, *F*(1,47) = 2.07, *p* = 0.16, η² = 0.02, BF_10_ = 0.06 (see Table 7 for recognition means and SDs).

**Table 7:** Recognition accuracy mean and SD scores in Experiment 3.

Condition	Mean	SD
Control	8.77	1.94
Interference	8.83	1.81
Reminder	9.21	1.74
Reminder + Interference	8.46	1.81

### Discussion

The reminder format employed in Experiment 3 exhibited evidence of being effective in improving recall accuracy. Furthermore, the change in phrasing for the interference manipulation seemingly led to a decline in recall accuracy for the reminder + interference combined condition compared to Experiment 2. Nevertheless, this was not sufficient to elicit any meaningful significant differences when compared to recall accuracy for the control and interference conditions, showing that the impact of using a reminder plus interference had little effect on recall accuracy, as opposed to the expected results according to the claim that reminders would engender reconsolidation interference.

## General discussion

Across three studies, we attempted to elicit behavioural reconsolidation interference effects in declarative memory using a within-subjects paradigm. This was employed to exclude the possibility of different retrieval strategies being used across separate reminder and interference conditions. Experiment 1 found no impact of reminder-potentiated interference on associative recognition memory, response times, or assays of recollection for deeply encoded object-picture pairs. Since even associative recognition might be conducted using familiarity judgments rather than recollection, and therefore might be less susceptible to the manipulation, different types of stimuli and retrieval were used in the following studies. Experiments 2 and 3 presented participants with stories as stimuli for all conditions, and assessed free recall success as a primary measure, followed up with yes/no questions adding a recognition component. Nonetheless, providing a reminder before introducing retroactive interference did not impair memory compared to simple interference in any of these paradigms. In other words, across six experiments altogether—three testing memory for item recognition^22^, one testing associative recognition, and two testing free recall, we did not observe evidence for behavioural reconsolidation interference in within-subjects’ paradigms.

As noted above, previous research has found evidence for behavioural reconsolidation interference in humans. James et al^13^. showed that emotional memories for traumatic clips displayed to participants were impaired after they played the visuospatial video game Tetris. Similarly, Schwabe and Wolf^15^ found that when memorizing a new story after recalling neutral and emotional experiences from participants’ past, memory for neutral experiences were impaired. It was thought that similar evidence of reconsolidation interference could be found in the current studies, changing the paradigm to a within-subjects design.

There are a few possible explanations why behaviorally mediated reconsolidation interference was not found in our three paradigms (item recognition, associative recognition, free recall) while being reported in prior studies. One reason could be the presentation of stimuli across the days. In Experiments 2 and 3, for example, participants were asked to think about the story from day one during the conditions that contained a reminder, but that reminder in itself might not have engendered lability necessary to engender interference. Some prior research has shown that prediction error may be key to the destablization of the memory^19,29^. Reminders that present similar information, but then change what is anticipated by the participant tend to be the most effective^18,30^. However, the ideal amount of prediction error to induce destabilizing lability is ambiguous^31^. It could be that only mentioning the story theme is insufficient to generate prediction error, and the stories should have been the same, or at least very similar, up to a certain point, before presenting new information (an interference) towards the end of the story, promoting prediction error and increasing the chance of effective interference.

It has also been shown that reconsolidation interference may be stronger in memories over one week old, in comparison to one day old^23^. However, this has not been found for all studies, as many have identified reconsolidation interference for memories that were only 24 h old^13,32^.

Another reason for the absence of reconsolidation interference found could be due to the lack of emotional valence of stimuli employed in the current studies. Previous research has shown successful evidence of reconsolidation interference, particularly when fear or trauma is used^2,13^, but this may also depend on participant’s specific traits also. For example, those with higher trait anxiety have poorer reduction of fear when pharmacologically inducing reconsolidation interference using propranolol^7^.

Regardless of these extraneous variables, there have still not been ample examples of behavioural reconsolidation interference for studies that have not used emotional stimuli. Earlier research showed the possibility of this effect, but in practice reported intrusions of interference material in the target material^33^. This may not specifically identify reconsolidation interference, but rather source memory confusion (i.e., the target memory has been reconsolidated, but the interference material is also thought to be a part of the encoding context). Further evidence for this is described by Hupbach et al.,^34^ stating that the intrusions tend to be identified only one-way, i.e., that intrusions from the target materials to the interference material are not observed.

In conclusion, the studies herein reported that it is at least very challenging to find the proper manipulation to engender reconsolidation interference in declarative memories using reminder-potentiated behavioural interference. However, there is always the possibility that further research will find a combination of manipulations under which such erasure of consolidated memories will be obtained. This remains important in the current field as cognitive-behavioural methods may still have the ability to modify and erase unwanted memories, offering value for updating learning, retraining, and therapeutic interventions.

## Methods

### Experiment 1

#### Participants

Participants were 40 young healthy adults (30 females, *M*_age_ = 24.18, SD = 3.78) who participated for academic credit required as part of their course of studies. This followed the sample size used in the studies in Levy et al.^22^ which serve as the basis for this experiment. Participants self-reported no psychiatric or neurological disorders. Participants had either normal or corrected to normal vision. Ethical approval was obtained from the Reichman University/Interdisciplinary Center Herzliya human participants research ethics committee, and participants provided signed informed consent before beginning the experiment. Participants were randomly assigned to one of four counterbalancing conditions in order to determine which picture pairs they would view in each of the three conditions on day two (reminder, interference, reminder + interference; see procedure for an explanation of conditions).

#### Materials

Three hundred object pictures from the Bank of Standardized Stimuli (BOSS^35^) were used as the stimuli for this experiment. Three lists of 100 pictures each were constructed for use as either A, B, or C stimuli (see procedure). Four of each list were used as practice for participants, leaving 96 object pictures in each list. The pictures constituted recognizable objects (such as a table, jar of honey, etc.) and were edited within Microsoft Paint to be comparable in detail, size (200 × 200 pixels) and resolution. The stimuli appeared on a white background and were presented in their original colours. Four practice pairs and 96 critical pairs and a complementary interference stimulus were randomly created from unrelated images, one from each list, but kept consistent across participants.

#### Procedure

E-Prime 3 was used to collect data from the first 12 participants in the laboratory. Due to COVID-19 lockdown restrictions, E-Prime Go was used to collect the following 28 participants, who performed the experiments at home while being supervised by an experimenter. As well as the experimenter being present at the time of all online data collection, participants were also instructed to be in a quiet place, without distractions and without any additional devices that could interrupt their concentration. The experiment was originally programmed for fixed display width (640 pixels) and height (480 pixels), but under the remote conditions this might have led to different display resolutions depending on participants’ screen settings. Whilst this may have differed across monitors, the displays would have been consistent across conditions for each participant. The experiment took place over three consecutive days at similar times of the day for each participant.

##### Day one.

Participants viewed 96 semantically unrelated object-picture pairs, composed of a cue picture (A; to the left of the screen) and a target picture (B; to the right of the screen). Participants were instructed to create a personal association between the two pictures, in order to ensure deep encoding. Participants were given a practice trial before the experiment began for the researchers to ensure the associations were appropriate. Furthermore, timing of the experiment was self-paced (up to a maximum of 15 s per pair) to allow deep associations to be made for each pair (mean encoding trial time = 11328 ms, SD = 3263 ms). To minimize fatigue, participants were given a 30 s rest break after encoding every 32 picture pairs.

##### Day two.

Twenty-four picture pairs were allocated to the encoding only (control) condition, and so were not shown on day two. For the reminder phase, participants were shown 48 of the picture pairs that they had previously seen on day one. Each pair had a presentation duration of 1500 ms. After viewing these picture pairs, they were given a three-minute rest break, after which the interference phase began. Following the break, participants were shown a further 48 picture pairs, combining the originally encoded cue (A) with a new target picture (C). Half of the cues had been seen recently during the reminder phase and half had only been seen on day one. This ensured that four conditions were established: an encoding only (control) where 24 pairs were only presented on day one; a reminder condition where 24 pairs had been learnt on day one and had been reminded on day two; an interference condition where 24 pairs had been learnt on day one and interfered with on day two; and finally, a reminder-interference combined condition where 24 pairs had been learnt on day one and had been reminded and, shortly after, interfered with on day two. As noted above, the assignment of each pair to one of these four conditions was counterbalanced across participants.

##### Day three

Participants were shown 96 triads of pictures containing the cue picture (A) in the upper area of the screen and a correct and incorrect target picture displayed side by side below the cue. Only one of the two target pictures was the correct target (B) that appeared with the cue on Day 1, whereas the second target picture (also B) had appeared alongside a different cue. Participants performed a forced choice recognition test where they had to select the correct picture that appeared with the cue on day one. Location of the correct target picture was counterbalanced so it would appear on each side of the screen 50% of the time. Participants selected the correct target image by pressing the right or left arrow keys. Finally, they were asked to indicate whether they ‘remembered’ the context of the picture pair (the association they made during encoding) or whether they just ‘knew’ that the pairs appeared together (without recalling the context). Specifically, participants were told to select ‘Remember’ if they had memory for the associated pair and context specific details about the memory, or ‘Know’ if they just remembered that the objects appeared together. Response time and accuracy were recorded for the recognition task.

### Experiment 2

#### Design

The study employed a 2 (Reminder/No Reminder) × 2 (Interference/No interference) repeated measures design with recall and recognition scores as DVs. Again, this led to four conditions under which participants were tested: control (no reminder, no interference), reminder only, interference only and reminder with interference.

#### Participants

Participants were 52 young healthy adults (43 females, *M*_age_ = 23.25, SD = 1.69) who participated for academic credit required as part of their course of studies. A slightly larger sample size than for Experiment 1 was employed to counter the lower amount of data points in this paradigm in comparison to the prior study. Participants self-reported no psychiatric or neurological disorders and had normal or corrected to normal vision. Ethical approval was obtained from the Reichman University/Interdisciplinary Center Herzliya human participants research ethics committee, and participants provided signed informed consent before beginning the experiment. Participants had not taken part in the preceding experiment.

#### Materials and procedure

**Fig. 5:**
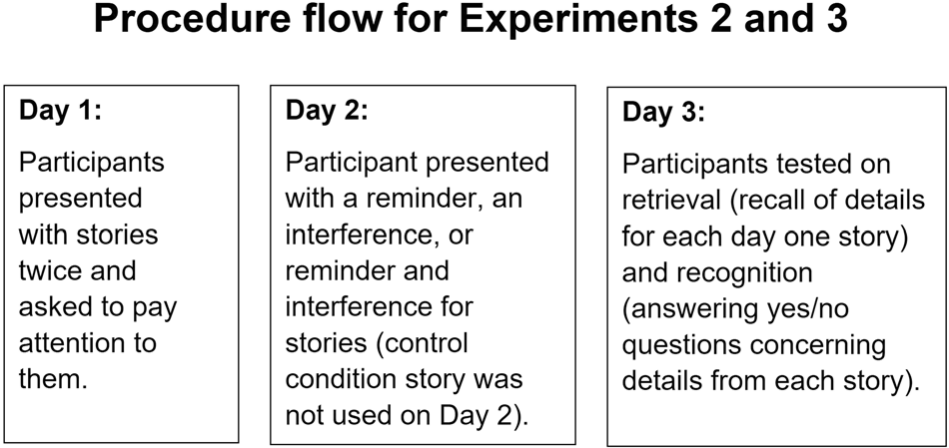
Procedure flow for Experiments 2 and 3.

The study took place online and was conducted through Zoom across three consecutive days (Fig. 5):

##### Day one.

Participants were presented with four separate stories on different topics, created by the researchers, which were based on stories included in the Logical Memory subtest of the Wechsler Memory Scale, fourth edition (WMS-IV^36^). This subtest is designed to measure immediate and delayed recall and delayed recognition, and so was appropriate for the current study. Two stories were created for each topic; one for the original presentation and one to be used as an interference. The stories were written about a robbery, the weather, road safety and farm animals. Participants are tested in the same way as the subtest in the WMS-IV by recalling as many details as possible, followed by a yes/no recognition test (see day three).

The stories were serially presented both visually (on slides) and auditorily (voice recording), to engender sufficiently strong initial encoding, given that the critical test would only be conducted 48 h later (as opposed to 30 m in the standard WMS-IV framework). For the written presentations, each sentence was presented individually every five seconds and the recordings were between roughly 30–40 s each. One story of each theme was assigned to one of the reminder/interference conditions, counterbalanced across participants. Once day one activities were completed, participants were then invited back to continue 24 h later.

##### Day two.

Participants were presented with information concerning three of the four conditions (there was no additional information for the control condition, as no reminder or interference task was presented for its story). For the reminder task, participants were given as a reminder the title assigned to the story on day one, and asked to think about that story for 30 s. For the interference condition, participants were presented (in the same way as on day one) with a new story which was based on the same topic as the interference-condition story from day one. Finally, for the reminder and interference condition, participants were given the title of this story from day one and asked to remember it for 30 s, followed by being presented with a new story on the same topic. The time between presentations was kept brief, as Levy et al.^22^ included two studies with a delay of 6–10 min (Study 1 and 3) and one without a substantial delay (study 2) but found no effect of reconsolidation interference for either delay length. The order of presentation of conditions was also counterbalanced across participants. Between each condition on day two, picture searches (where participants had to find a hidden item within an image) were presented for three minutes acting as a wash-out activity, in order to prevent details from the previous story (either presented or remembered) carrying over to the next condition.

##### Day three.

Participants were first asked to recall as many details as they could from each story presented on day one. This was asked in the same order as the stories were presented on day one, and participants were given as much time as needed to recall all details. A uniform checklist of 25 details for each story was marked by the researcher in real time. After testing their recall for each condition, participants were then asked 15 yes/no questions about each story to test their recognition. Again, these were asked in the same order as the stories were presented on day one. Once completed, participants were debriefed and thanked for their time.

### Experiment 3

#### Design

The study employed a 2 (Reminder/No Reminder) × 2 (Interference/No interference) repeated measures design with recall and recognition scores as DVs. Again, this led to four conditions that participants were tested on: control (no reminder, no interference), reminder only, interference only and reminder with interference.

#### Participants

Participants were 48 young healthy adults (43 females, *M*_age_ = 23.46, SD = 1.41) who participated for academic credit required as part of their course of studies. Participants self-reported no psychiatric or neurological disorders and had normal or corrected to normal vision. Ethical approval was obtained from the Reichman University/Interdisciplinary Center Herzliya human participants research ethics committee, and participants provided signed informed consent before beginning the experiment. Participants had not taken part in the two preceding experiments.

#### Materials and procedure

The materials and procedure were similar to the previous study, except for some changes made on day two. When participants received a reminder, instead of being given 30 s to think about the story from the previous day, they were given three facts about the story from day one. This was done to reduce the amount of time participants thought about the original stories, and to remind them of specific points from the original stories to trigger their memory of them. The facts used were taken from the yes/no recognition questions, so these three questions were not asked on day three, bringing the total number of recognition questions asked from 15 to 12.

To increase the strength of the interference, the researcher emphasized the title of the story. For example, before presentation of the story, the following would be said to the participant; “now you are going to see another story about [story title]” and after presentation of the story the following would be said “so that was another story about [story title]”. This was done in order to ensure that the participant understood that the story now presented was related to and could be processed as similar to the story heard on day one.

### Reporting summary

Further information on research design is available in the Nature Research Reporting Summary linked to this article.

## Supplementary information

Reporting Summary

## Data Availability

The full dataset of the findings reported in this article is available at https://osf.io/dy4e8/.

## Code availability

No proprietary code was written for these studies.
