# Supplementary material for: Behavioural reconsolidation interference not observed in a within-subjects design
Source: NPJ Sci Learn. 2022 Oct 11;7:26. doi: 10.1038/s41539-022-00143-w (PMC9553081; doi:10.1038/s41539-022-00143-w)
Supplement: Supplementary file 1 — Reporting Summary [file 41539_2022_143_MOESM1_ESM.pdf]

nature portfolio

Corresponding author(s): Daniel A. Levy

Last updated by author(s): Jul 24, 2022

Reporting Summary

Nature Portfolio wishes to improve the reproducibility of the work that we publish. This form provides structure for consistency and transparency in reporting. For further information on Nature Portfolio policies, see our Editorial Policies and the Editorial Policy Checklist.

Statistics

For all statistical analyses, confirm that the following items are present in the figure legend, table legend, main text, or Methods section.

n/a	Confirmed	
☐	☒	The exact sample size (*n*) for each experimental group/condition, given as a discrete number and unit of measurement
☐	☒	A statement on whether measurements were taken from distinct samples or whether the same sample was measured repeatedly
☐	☒	The statistical test(s) used AND whether they are one- or two-sided<br>*Only common tests should be described solely by name; describe more complex techniques in the Methods section.*
☒	☐	A description of all covariates tested
☐	☒	A description of any assumptions or corrections, such as tests of normality and adjustment for multiple comparisons
☐	☒	A full description of the statistical parameters including central tendency (e.g. means) or other basic estimates (e.g. regression coefficient) AND variation (e.g. standard deviation) or associated estimates of uncertainty (e.g. confidence intervals)
☐	☒	For null hypothesis testing, the test statistic (e.g. *F*, *t*, *r*) with confidence intervals, effect sizes, degrees of freedom and *P* value noted<br>*Give P values as exact values whenever suitable.*
☐	☒	For Bayesian analysis, information on the choice of priors and Markov chain Monte Carlo settings
☒	☐	For hierarchical and complex designs, identification of the appropriate level for tests and full reporting of outcomes
☐	☒	Estimates of effect sizes (e.g. Cohen's *d*, Pearson's *r*), indicating how they were calculated

Our web collection on statistics for biologists contains articles on many of the points above.

Software and code

Policy information about availability of computer code

Data collection	E-Prime 3.0 and E-Prime Go
Data analysis	SPSS

For manuscripts utilizing custom algorithms or software that are central to the research but not yet described in published literature, software must be made available to editors and reviewers. We strongly encourage code deposition in a community repository (e.g. GitHub). See the Nature Portfolio guidelines for submitting code & software for further information.

Data

Policy information about availability of data

All manuscripts must include a data availability statement. This statement should provide the following information, where applicable:

- Accession codes, unique identifiers, or web links for publicly available datasets
- A description of any restrictions on data availability
- For clinical datasets or third party data, please ensure that the statement adheres to our policy

The full dataset of the findings reported in this article is available at https://osf.io/dy4e8/ .

Human research participants

Policy information about studies involving human research participants and Sex and Gender in Research.

Reporting on sex and gender	Participants of both sexes were included, and the distributions of sex of participants in each experiment are reported. Sex was not considered in the study design. Sex was self-reported. The source data uploaded to public repository includes non-aggregated individual data; consent was not requested for sharing individual-level data. Sex-based analyses were not performed, as in prior research there is no indication that sex-based factors are relevant to the research question.
Population characteristics	Participants were self-reportedly healthy, ages 18-30. No other diagnostic or genotypic information was collected.
Recruitment	Participants were all active undergraduate students. They were recruited through an institutional student-participation website, and received academic credit or money in return for participation. Since the studies were designed as within-participant paradigms, self-selection bias was not considered relevant.
Ethics oversight	Reichman University research ethics committee

Note that full information on the approval of the study protocol must also be provided in the manuscript.

Field-specific reporting

Please select the one below that is the best fit for your research. If you are not sure, read the appropriate sections before making your selection.

☐ Life sciences ☒ Behavioural & social sciences ☐ Ecological, evolutionary & environmental sciences

For a reference copy of the document with all sections, see nature.com/documents/nr-reporting-summary-flat.pdf

Behavioural & social sciences study design

All studies must disclose on these points even when the disclosure is negative.

Study description	Quantitative within-participants design
Research sample	140 Reichman University undergraduate students ages 18-30, 116 F (representing the sampling population distribution)
Sampling strategy	Volunteers sampling was used. Participants were recruited through an institutional student-participation website, and received academic credit or money in return for participation.<br>Study 1 followed the sample size used in Levy et al. (2018) in order to compare to this previous research. As we did not have prior estimates of effect sizes for studies 2 and 3, we decided to use a slightly larger sample size to counter the lower amount of data points in comparison to study 1 to use slightly larger sample size because we had fewer data points.
Data collection	Data was collected either in the lab where only the researcher and participant were present, online where the participant completed the study alone, or over Zoom where only the participant and researcher were present on the meeting.<br>E-Prime 3.0 and E-Prime Go were used for data collection in study 1 and verbal report with manual scoring was used in studies 2 and 3.
Timing	Study 1: October 2019 - September 2020<br>Study 2: January 2021 - July 2021<br>Study 3: October 2021 - January 2022
Data exclusions	Two participants data was excluded in study 1 as there were technical errors in the E-Prime Go program functionality.
Non-participation	No participants dropped out from the study.
Randomization	Participants were not allocated into separate groups as the design was within-subjects

Reporting for specific materials, systems and methods

We require information from authors about some types of materials, experimental systems and methods used in many studies. Here, indicate whether each material, system or method listed is relevant to your study. If you are not sure if a list item applies to your research, read the appropriate section before selecting a response.

Materials & experimental systems

n/a	Involved in the study
☒	☐ Antibodies
☒	☐ Eukaryotic cell lines
☒	☐ Palaeontology and archaeology
☒	☐ Animals and other organisms
☒	☐ Clinical data
☒	☐ Dual use research of concern

Methods

n/a	Involved in the study
☒	☐ ChIP-seq
☒	☐ Flow cytometry
☒	☐ MRI-based neuroimaging
